# Baseline anemia predicts a poor prognosis in patients with non-small cell lung cancer with epidermal growth factor receptor mutations: a retrospective study

**DOI:** 10.1186/s12890-022-02158-w

**Published:** 2022-10-17

**Authors:** Jingwen Wei, Jing Xiang, Yue Hao, Jinfei Si, Wenxian Wang, Fangyin Li, Zhengbo Song

**Affiliations:** 1 Department of Clinical Trial, The Cancer Hospital of the University of Chinese Academy of Sciences (Zhejiang Cancer Hospital), No. 1 Banshan East Road, Gongshu District, Hangzhou, 310022 China; 2 Wenzhou Medical University, Wenzhou, China; 3 The Second Clinical Medical College of Zhejiang, Chinese Medical University, Hangzhou, China; 4 Department of Medical Oncology, The Cancer Hospital of the University of Chinese Academy of Sciences (Zhejiang Cancer Hospital), Hangzhou, China; 5 Department of Urology, The Cancer Hospital of the University of Chinese Academy of Sciences (Zhejiang Cancer Hospital), No. 1 Banshan East Road, Gongshu District, Hangzhou, 310022 China

**Keywords:** Anemia, Epidermal growth factor receptor, Epidermal growth factor receptor tyrosine kinase inhibitors, Non-small cell lung cancer

## Abstract

**Background:**

Anemia is relatively common in cancer patients, and baseline anemia is associated with poor survival in patients with non-small cell lung cancer (NSCLC). However, there is a lack of large-sample studies of patients with NSCLC with epidermal growth factor receptor (EGFR) mutations.

**Methods:**

We retrospectively analyzed anemia‑related data for patients with NSCLC and *EGFR* mutations who were admitted to Zhejiang Cancer Hospital from January 2013 to June 2019 and treated with targeted therapy. The patients’ clinicopathological features were evaluated by χ^2^ tests and the relationships between clinical characteristics and prognosis were investigated using Kaplan–Meier and multivariate Cox regression analyses.

**Results:**

A total of 2,029 patients treated with EGFR-tyrosine kinase inhibitors (TKIs) were finally enrolled in this study, of whom 24.6% had baseline anemia. Patients without baseline anemia had longer median overall survival (OS) than patients with baseline anemia (36.10 vs. 29.10 months, *P* = 0.001), and patients with grade < 2 anemia had longer median OS than those with grade ≥ 2 anemia (35.00 vs. 25.10 months, *P* < 0.001). Multivariate analyses identified baseline anemia as a factor predicting a poor prognosis in terms of OS in patients with *EGFR* mutations.

**Conclusions:**

Baseline anemia is a significant factor predicting a poor prognosis in terms of OS in patients with NSCLC and *EGFR* mutations treated with targeted therapy. A higher grade of baseline anemia may also be related to shorter OS. And a higher risk of *EGFR*-mutated patients who had received targeted therapy could also be observed.

## Background

Lung cancer is the most commonly diagnosed cancer and the leading cause of cancer-related death [1, 2]. There are two main histological forms of lung cancer: non-small cell lung cancer (NSCLC), accounting for 85% of patients, and small cell lung cancer, accounting for the remaining 15% of patients [3, 4]. The World Health Organization estimates that lung cancer death rates will continue to rise worldwide, particularly in Asia, in line with increasing tobacco use [5].

Epidermal growth factor receptor (EGFR) gene mutations are common in patients with NSCLC. About one-third of patients with NSCLC carry *EGFR* mutations, with higher incidences in Asians, women, non-smokers, and adenocarcinoma patients [6, 7]. EGFR-targeted therapy has been an important step forward in the treatment of patients with *EGFR* mutations. However, although *EGFR* mutations provide a promising biomarker for patients with lung cancer treated with EGFR-tyrosine kinase inhibitors (TKIs), around 20% of patients with *EGFR* mutations fail to respond to these agents [8, 9]. Clinicians thus need to find effective factors to identify patients likely to benefit from treatment with EGFR-TKIs.

Cancer-related anemia (CRA) is relatively common in cancer patients and represents a multifactorial problem, with its severity potentially affected by immune, nutritional, and metabolic components [10]. Although the underlying mechanisms of CRA are unclear, it is thought to be caused directly by tumor suppression of hematopoiesis through bone marrow infiltration or via the production of cytokines leading to iron sequestration [11]. Previous studies have shown that CRA may be related to the activation of cytokines such as interferon-γ, interleukin-1, and tumor necrosis factor, which may inhibit the production of endogenous erythropoietin, impair iron use, and reduce the proliferation of erythrocyte precursors [12, 13]. CRA also exacerbates tumor hypoxia, which in turn increases tumor-cell tolerance and resistance to radiotherapy and chemotherapy, thus affecting clinical treatment efficacy and patient survival [14–16]. However, the impact of anemia on targeted therapy is still unclear.

Pretreatment anemia is associated with a poor prognosis in various malignant tumors, and this relationship has been a focus of clinical attention. A study of 147 patients with early-stage NSCLC treated with stereotactic body radiation therapy found that pretreatment anemia was a predictive factor of poor overall survival (OS), which is likely to reflect disease progression [17]. Tomita et al. [18] measured preoperative white blood cell counts, hemoglobin (Hb) levels, and platelet counts in 289 consecutive patients with NSCLC who had undergone surgical resection and found that the 5-year survival rates of patients with leukocytosis, anemia, and thrombocytopenia were only 25.0%, which was significantly worse than that of patients with normal blood cell counts (78.23%). Pre-treatment anemia is thus associated with a poor prognosis in patients with lung cancer. However, few large-sample studies have been conducted in patients with NSCLC and *EGFR* mutations treated with EGFR-TKIs. We therefore conducted a retrospective study of 2,029 patients with stage IV *EGFR*-mutated NSCLC who received EGFR-TKI therapy to investigate the prognostic value of baseline anemia and anemia grade in these patients. This represents the largest study of the importance of anemia in *EGFR*-mutated NSCLC.

## Methods

### Clinical data

We retrospectively analyzed anemia‑related data for patients with stage IV *EGFR*-mutated NSCLC who were admitted to Zhejiang Cancer Hospital, Hangzhou, China, from January 2013 to June 2019. A total of 2,029 patients were eligible for the study. The inclusion criteria were: i) pathologically confirmed stage IV NSCLC with *EGFR* mutations; ii) age > 18 years; iii) complete clinical data and follow‑up information; and iv) subsequent receipt of EGFR-TKIs. The exclusion criteria were: i) basic hematological diseases and ii) chronic nephropathy. Clinical staging was based on the 8th edition of the Tumor, Node, Metastasis staging system [19]. We extracted the following data from the patients’ medical records: patient demographics, smoking history, stage of lung cancer, pathological type of tumor, *EGFR* mutation subtype, brain metastasis, bone metastasis, antitumor treatment options, C-Reactive protein (CRP), baseline Hb content, and grading. *EGFR* mutation subtypes were detected by next-generation sequencing or polymerase chain reaction. In the study, EGFR-TKIs used were gefitinib (250 mg per day orally), erlotinib (150 mg per day orally), icotinib (125 mg three times per day orally), afatinib (40 mg per day orally) or osimertinib (80 mg per day orally). The follow‑up period lasted until October 2019.The protocol was approved by the institutional review board of Zhejiang Cancer Hospital. The study was carried out in accordance with the guidelines of the Helsinki Declaration (as revised in 2013) and the need for individual consent for this retrospective analysis was waived.

### Grading of anemia

Baseline anemia was defined as a Hb level < 120.0 g/l for males and < 110.0 g/l for females at their first visit to the hospital. No patients had received radiotherapy, chemotherapy, targeted therapy, or immunotherapy. According to the National Cancer Institute criteria, anemia was categorized into five grades according to Hb level: grade 0, normal Hb level; grade 1 (mild anemia), males 100–120 g/l, females 100–110 g/l; grade 2 (moderate anemia), 80–100 g/l; grade 3 (severe anemia), 65–80 g/l; and grade 4 (life-threatening anemia), < 65 g/l [20]. In this study, patients were divided into non-anemic (n = 1,529) and anemic (n = 500) groups based on their baseline Hb levels.

### Statistical analysis

The data were analyzed using Statistical Package for the Social Sciences (SPSS Inc., Chicago, IL, USA) version 25.0 software. Quantitative variables were presented as mean ± standard deviation. Differences in demographic and clinical characteristics between the anemic and non-anemic groups were analyzed by Pearson’s χ^2^ test. Survival analysis was conducted using the Kaplan–Meier method and comparisons were made using the log-rank test. Hazard ratio (HR) and 95% confidence interval (CI) were calculated by Cox proportional hazard regression analysis. A *P* value < 0.05 was regarded as statistically significant.

## Results

### Patient characteristics

A total of 2,029 eligible patients with *EGFR* mutation-positive stage IV NSCLC were included in the study, all of whom received subsequent targeted therapy. Based on their baseline Hb levels, 1,529 (75.4%) and 500 (24.6%) patients were included in the non-anemic and anemic groups, respectively. The average Hb level in the 500 anemic patients was 102.45 ± 11.81 g/l. According to the National Cancer Institute scoring system, 328 patients (65.6%) had mild anemia, 148 (29.6%) had moderate anemia, 22 (4.4%) had severe anemia, and two (0.4%) had life-threatening anemia (Table 1). The demographic and clinical characteristics of the patients are summarized in Table 2. Baseline anemia was significantly less common in male than female patients (49.4% vs. 50.6%, *P* = 0.024). *EGFR*-mutation type also differed significantly between anemic and non-anemic patients (*P* = 0.014): the proportions of exon 19 deletions (44.6% vs. 45.3%) and L858R point mutations (41.0% vs. 44.9%) were lower while the rate of other mutation subtypes (14.4% vs. 9.8%) was higher in the anemic group compared with the non-anemic group. The incidence of baseline anemia was significantly lower in patients without bone metastasis than in patients with bone metastasis (22.5% vs. 26.6%, *P* = 0.034). There were no significant differences in age, ECOG PS, smoking history, pathological type, brain metastasis, CRP or EGFR-TKI between the two groups (*P* > 0.05).

**Table 1:** Frequency of first-admission baseline cancer-related anemia in the current study

Grade	Hb (mean ± SD, g/l)	Number
0	134.22 ± 13.18	1529
1	109.27 ± 5.98	328
2	92.20 ± 5.62	148
3	73.82 ± 4.40	22
4	58.00 ± 1.41	2
1–4	102.45 ± 11.81	500

*Hb* Hemoglobin, *SD* Standard deviation

**Table 2:** Demographic and clinical characteristics of 2,029 patients with NSCLC

Characteristic	Patients (n, %)	Without anemia (n, %)	With anemia (n, %)	*P* value
Sex				0.024
Male	914 (45.0)	667 (43.6)	247 (49.4)	
Female	1115 (55.0)	862 (56.4)	253 (50.6)	
Age (years)				0.316
≤ 65	1408 (69.4)	1070 (70.0)	338 (67.6)	
> 65	621 (30.6)	459 (30.0)	162 (32.4)	
ECOG PS				0.113
0–1	1569 (77.3)	1190 (77.8)	379 (75.8)	
2	351 (17.3)	252 (16.5)	99 (19.8)	
Unknown	109 (5.4)	87 (5.7)	22 (4.4)	
Smoking history				0.074
No	1313 (64.7)	1006 (65.8)	307 (61.4)	
Yes	716 (35.3)	523 (34.2)	193 (38.6)	
Pathological type				0.074
Adenocarcinoma	1839 (90.6)	1400 (91.6)	439 (87.8)	
Squamous cell carcinoma	178 (8.8)	122 (8.0)	56 (11.2)	
Large cell carcinoma	7 (0.3)	4 (0.3)	3 (0.6)	
Adenosquamous carcinoma	5 (0.2)	3 (0.2)	2 (0.4)	
*EGFR* mutation subtype				0.014
Exon 19 deletion	916 (45.1)	639 (45.3)	223 (44.6)	
L858R	891 (43.9)	686 (44.9)	205 (41.0)	
Other	222 (10.9)	150 (9.8)	72 (14.4)	
Brain metastasis				0.535
No	1350 (66.5)	1023 (66.9)	327 (65.4)	
Yes	679 (33.5)	506 (33.1)	173 (34.6)	
Bone metastasis				0.034
No	972 (47.9)	753 (49.2)	219 (43.8)	
Yes	1057 (52.1)	776 (50.8)	281 (56.2)	
Surgical history				0.092
No	1225 (80.1)	383 (76.6)	1608 (79.3)	
Yes	304 (19.9)	117 (23.4)	421 (20.7)	
Radiotherapy				0.016
No	1273 (62.7)	928 (64.2)	291 (58.2)	
Yes	756 (37.3)	547 (35.8)	209 (41.8)	
Chemotherapy				< 0.001
No	799 (39.4)	676 (44.2)	123 (24.6)	
Yes	1230 (60.6)	853 (55.8)	377 (75.4)	
EGFR-TKI				0.331
Gefitinib	709 (34.9)	552 (36.1)	157 (31.4)	
Erlotinib	31 (1.5)	24 (1.6)	7 (1.4)	
Icotinib	1234 (60.8)	915 (59.8)	319 (63.8)	
Afatinib	36 (1.8)	25 (1.6)	11 (2.2)	
Osimertinib	19 (0.9)	13 (0.9)	6 (1.2)	
CRP elevated				0.638
No	1188 (58.6)	900 (58.9)	288 (57.6)	
Yes	841 (41.4)	629 (41.1)	212 (42.4)	
Grade of anemia				< 0.001
< 2	1857 (8.4)	1529 (100)	328 (65.6)	
≥ 2	172 (24.6)	0 (0.0)	172 (34.4)	

*CRP* C-Reactive protein, *EGFR* Epidermal growth factor receptor, *ECOG PS* Eastern Cooperative Oncology Group Performance status, *NSCLC* Non-small cell lung cancer

### Effects of baseline anemia and grade on patient survival

**Fig. 1:**
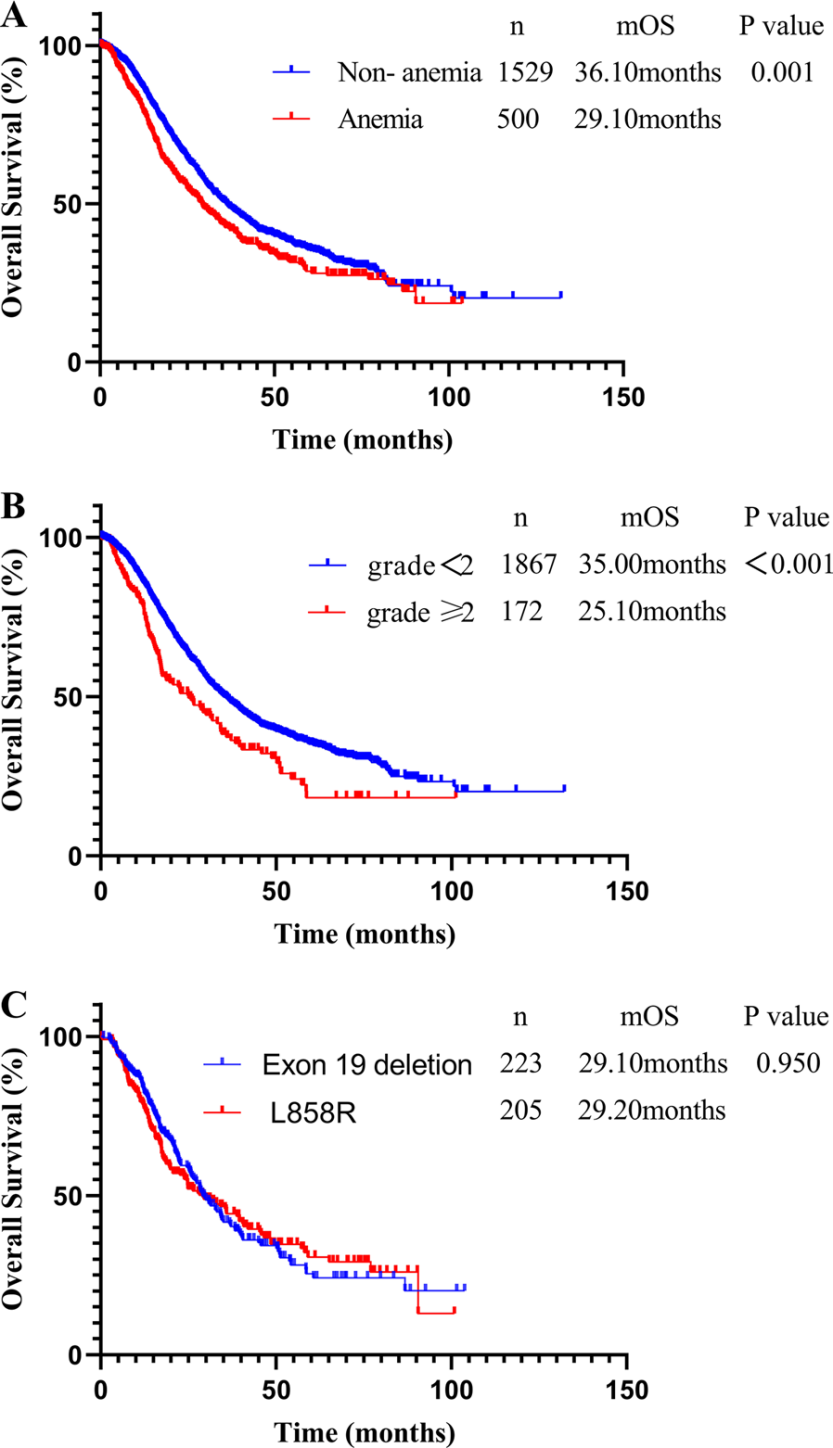
Kaplan–Meier curves of overall survival (OS). OS in **A** patients with and without anemia, **B** patients with anemia grade < 2 and grade ≥ 2, and **C** patients with *EGFR* exon 19 deletions and L858R point mutations

Patients with anemia had a shorter median OS than patients without anemia [29.10 (95% CI, 25.05–33.15) months vs. 36.10 (95% CI, 33.07–39.13) months, *P* = 0.001] (Fig. 1A). Patients were divided into two groups according to the grade of anemia (grade < 2 and grade ≥ 2). Higher baseline anemia grade was associated with a shorter OS and poorer prognosis: patients with grade < 2 anemia had significantly longer median OS than those with grade ≥ 2 anemia [35.00 (95% CI 32.45–37.55) months vs. 25.10 (95% CI, 17.03–33.14) months, *P* < 0.001] (Fig. 1B). In addition, among anemic patients with *EGFR* mutations, the median OS durations were similar in patients with exon 19 deletions and those with L858R point mutations [29.10 (95% CI, 24.45–33.76) vs. 29.20 (95% CI, 21.12–37.28) months, respectively, *P* = 0.950] (Fig. 1C).

### Univariate and multivariate analyses of clinical features and prognosis

**Fig. 2:**
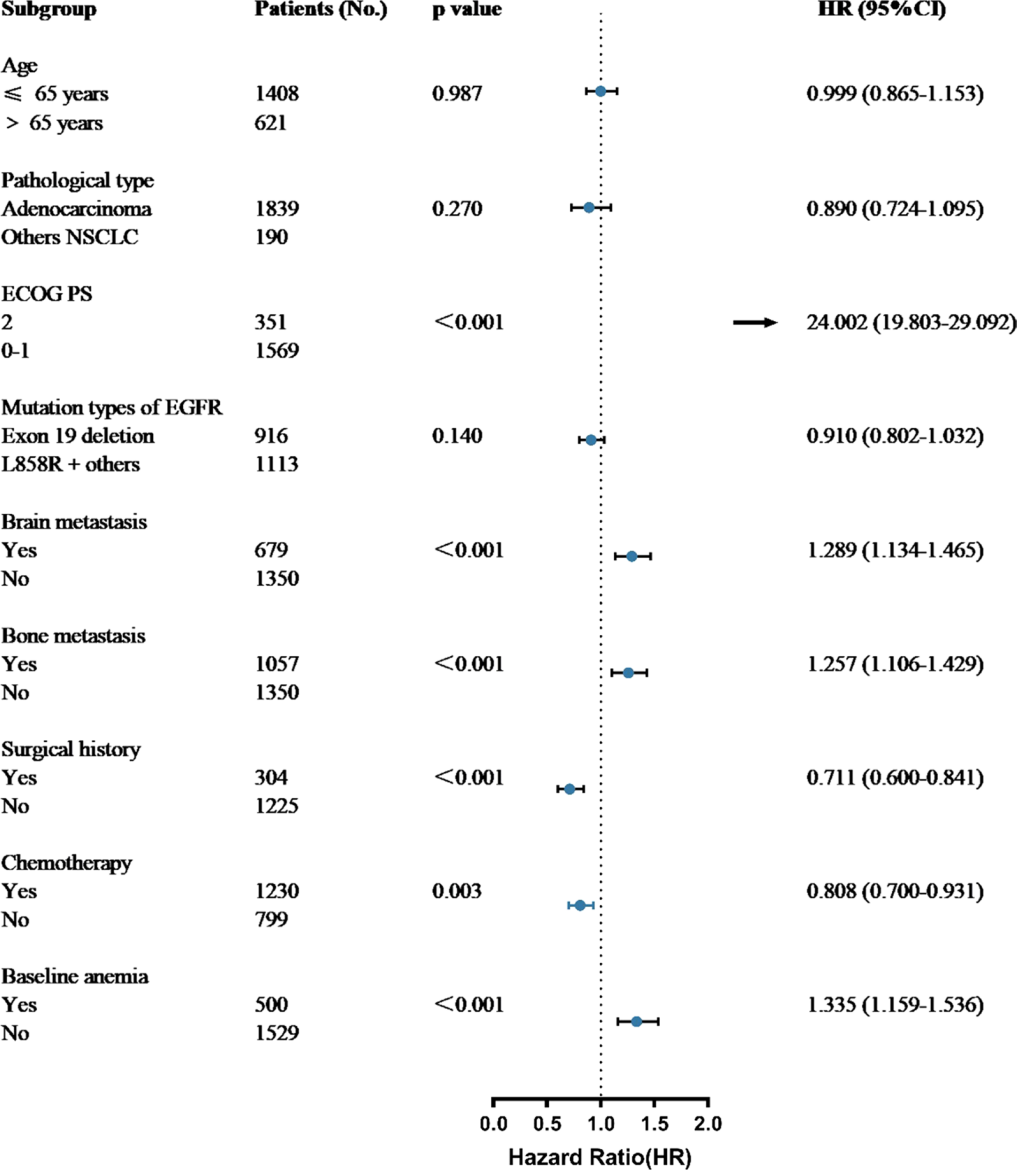
Forest plot of potential factors affecting overall survival in patients with *EGFR*-mutated NSCLC, using a Cox proportional hazards model. No.: number; NSCLC: non-small cell lung cancer; EGFR: epidermal growth factor receptor; HR: hazard ratio; CI: confidence interval

Univariate analysis showed that OS was significantly correlated with age, ECOG PS, pathological type, *EGFR-*mutation subtype, brain metastasis, bone metastasis, surgical history, chemotherapy, and baseline anemia (Table 3). In contrast, there was no significant correlation with sex, smoking history, or radiotherapy. Factors that were significant in univariate analysis were included in Cox multivariate regression analysis, which identified ECOG PS, brain metastasis, bone metastasis, surgical history, chemotherapy, and baseline anemia as independent prognostic factors for OS in patients with stage IV *EGFR*-mutated NSCLC who received targeted therapy. These results indicated that baseline anemia was associated with a poor prognosis in patients with NSCLC with *EGFR* mutations (Fig. 2).

**Table 3:** Univariate analysis of prognostic variables in patients with EGFR mutations receiving EGFR-TKIs

Characteristic	HR	95% CI	P value
Sex (female vs. male)	0.930	0.824–1.094	0.237
Age (≤ 65 years vs. > 65 years)	0.832	0.729–0.949	0.006
Pathological type (adenocarcinoma vs. others NSCLC)	0.794	0.649–0.972	0.025
ECOG PS (2 vs. 0–1)	24.454	20.244–29.539	< 0.001
Smoking history (yes vs. no)	1.001	0.882–1.136	0.986
EGFR-mutation subtype (Exon 19 deletion vs. L858R)	0.870	0.771–0.982	0.025
Brain metastasis (yes vs. no)	1.244	1.099–1.409	0.001
Bone metastasis (yes vs. no)	1.333	1.180–1.506	< 0.001
Operation History (yes vs. no)	0.621	0.528–0.731	< 0.001
Radiotherapy (yes vs. no)	0.908	0.802–1.028	0.129
Chemotherapy (yes vs. no)	0.802	0.704–0.913	0.001
Baseline anemia (yes vs. no)	1.256	1.098–1.436	0.001

*EGFR* Epidermal growth factor receptor, *ECOG PS* Eastern Cooperative Oncology Group Performance status, *NSCLC* Non-small cell lung cancer, *TKI* Tyrosine kinase inhibitor

## Discussion

We compared the clinical outcomes of patients with NSCLC harboring activating *EGFR* mutations who received targeted therapy. Baseline anemia was significantly correlated with prognosis in these patients, with OS decreasing in line with increasing anemia grade in patients with *EGFR* mutations treated with targeted therapy. To the best of our knowledge, the current study including 2,029 patients was the largest study of anemia in patients with *EGFR*-mutated NSCLC treated with targeted therapy. The aim of the study was to investigate the prognostic value of baseline anemia and anemia grade in these patients.

In the 2004 European Cancer Anaemia Survey report, 39% of cancer patients had pre-treatment anemia at the start of the survey [21]. In our study, 1,529 (75.4%) and 500 (24.6%) patients were classified as non-anemic and anemic, respectively. The mean Hb level among the 500 patients with baseline anemia was 102.45 ± 11.81 g/l, and most patients with baseline anemia had mild to moderate anemia, including 328 patients (65.6%) with mild anemia and 148 (29.6%) with moderate anemia, compared with only 22 (4.4%) with severe anemia and two (0.4%) with life-threatening anemia.

The present analysis identified baseline anemia as a factor associated with a poor prognosis in patients with NSCLC with *EGFR* mutations. Previous studies also showed that baseline anemia could affect patients’ clinical outcomes. An analysis [22] of 186 NSCLC patients with *EGFR* mutations treated with first-line tyrosine kinase inhibitors found that anemic patients had shorter median OS than non-anemic patients [24.83 (95% CI, 17.49–32.17) months vs. 42.10 (95% CI, 31.87–52.34) months, *P* = 0.031], and anemia [HR = 2.573 (95% CI, 1.12–5.90), *P* = 0.026] was the only independent factor predicting poor OS. Chen et al. [23] reported that baseline anemia and anemia grade were independent prognostic factors in patients with stage IV NSCLC. They found that patients without baseline anemia had longer OS than patients with baseline anemia (28.0 vs. 17.4 months, *P* < 0.001) and OS decreased with increasing anemia grade, with patients with grade 0 anemia having the longest OS (28.0 months) and patients with grade 3 and 4 anemia having the shortest OS (8.6 months). A meta-analysis of 23 studies by Liu et al. showed that preoperative anemia was associated with poor OS in lung cancer patients, and the risk of death was about 1.58 times higher in patients with preoperative anemia compared with patients without anemia [summarized HR = 1.58 (95% CI, 1.44–1.75)] [24]. Tanaka et al. [25] evaluated the prognostic significance of anemia in patients with NSCLC undergoing stereotactic body radiation therapy, and anemia was confirmed as the only significant factor in multivariate analysis (*P* = 0.025). Another study showed that anemia was not an independent predictor of short-term outcomes but was independently associated with significantly reduced survival in patients undergoing resection for lung cancer [26]. These studies demonstrated that anemia was associated with a poor prognosis in patients with NSCLC. However, the sample sizes of previous studies have been small, and few studies have been limited to patients with *EGFR* mutations who received targeted therapy.

In this study, we focused on the relationship between baseline anemia and prognosis in patients with *EGFR*-mutated NSCLC treated with targeted therapy. We found that male sex, and bone metastasis were significantly associated with a higher incidence of baseline anemia. In addition, the mutation subtypes varied between patients with and without anemia: the proportions of exon 19 deletions and L858R point mutations were lower in the anemia group compared with the non-anemic group, while the rate of other mutation subtypes was conversely higher in the anemic group. The rates of baseline anemia were also higher in patients > 65 years old, smokers, and patients with brain metastasis compared with the corresponding population, but the differences were not significant. Patients without baseline anemia had longer OS than patients with baseline anemia [36.10 (95% CI, 33.07–39.13) months vs. 29.10 (95% CI, 25.05–33.15) months, *P* = 0.001], and OS decreased with increasing baseline anemia grade, with OS being longer in patients with grade < 2 anemia compared with those with grade ≥ 2 anemia [35.00 (95% CI 32.45–37.55) months vs. 25.10 (95% CI, 17.03–33.14) months, *P* < 0.001]. Moreover, the median OS in patients with exon 19 deletions was similar to that in patients with L858R point mutations [29.10 (95% CI, 24.45–33.76) vs. 29.20 (95% CI, 21.12–37.28) months, *P* = 0.950]. Univariate and multivariate analyses found that ECOG PS, brain metastasis, bone metastasis, surgical history, chemotherapy, and baseline anemia were independent prognostic factors for OS in patients with stage IV *EGFR*-mutated NSCLC who received targeted therapy. These results indicate the prognostic value of baseline anemia in patients with NSCLC with *EGFR* mutations, thus highlighting the importance of the early treatment of anemia and the control of Hb levels in cancer patients, and suggesting that improving the management of anemia may also prolong patient survival.

This study had some limitations. Importantly, the primary data were obtained retrospectively, which may have affected the results, and this was also a single‑center study. In addition, control of other comorbid conditions may have been lacking, and baseline anemia could be a surrogate for other medical comorbidities, which may confound our results. However, as the largest study of anemia in patients with NSCLC with *EGFR* mutations, we believe that the results are of great significance and may help to guide the treatment of NSCLC in patients with *EGFR* mutations. However, more multicenter prospective studies are needed to confirm our results.

## Conclusions

This study demonstrated that baseline anemia and anemia grade were significantly correlated with prognosis in patients with NSCLC with *EGFR* mutations who received targeted therapy. These data suggest that routine measurement of Hb levels during cancer work-up may help to predict the prognosis in NSCLC patients with *EGFR* mutations.

## Data Availability

The datasets generated and/or analyzed during the current study are not publicly available due to limitations of ethical approval involving the patient data and anonymity but are available from the corresponding author on reasonable request.

## Abbreviations

CRA: Cancer-related anemia
EGFR: Epidermal growth factor receptor
TKI: Tyrosine kinase inhibitor
Hb: Hemoglobin
HR: Hazard ratio
CI: Confidence interval
NSCLC: Non-small cell lung cancer
OS: Overall survival
SD: Standard deviation
